# Fat-to-Muscle Ratios and the Non-Achievement of LDL Cholesterol Targets: Analysis of the Korean Genome and Epidemiology Study

**DOI:** 10.3390/jcdd8080096

**Published:** 2021-08-12

**Authors:** A-Ra Cho, Jun-Hyuk Lee, Yu-Jin Kwon

**Affiliations:** 1 Department of Family Medicine, Yongin Severance Hospital, Yonsei University College of Medicine, Yongin 16995, Korea; ARA1713@yuhs.ac; 2 Department of Family Medicine, Nowon Eulji Medical Center, Eulji University School of Medicine, Seoul 01830, Korea

**Keywords:** fat-to-muscle ratio, fat mass, muscle mass, low-density lipoprotein cholesterol, cardiovascular risk, cohort study

## Abstract

Maintaining optimal low-density lipoprotein (LDL) cholesterol levels is necessary to prevent cardiovascular disease (CVD). Excessive fat mass and decreased muscle mass are both associated with increased risks of developing dyslipidemia. Thus, we investigated the longitudinal relationship between the fat-to-muscle ratio (FMR) and the non-achievement of LDL cholesterol targets. We analyzed a total of 4386 participants aged 40–69 years from the Korean Genome and Epidemiology Study. FMR was defined as the ratio of total fat mass to total muscle mass, measured by bioelectrical impedance. The non-achievement of an LDL cholesterol target was defined as an LDL cholesterol level higher than the established target level according to individual CVD risk. The adjusted hazard ratios and 95% confidence interval for the incidence of non-achievement of LDL cholesterol targets for the sex-specific middle and highest tertiles vs. the referent lowest tertile of FMR were 1.56 (1.29–1.90) and 1.86 (1.47–2.31) in men and 1.40 (1.18–1.66) and 1.31 (1.06–1.62) in women after adjusting confounders. Our findings suggest that FMR, a novel indicator of the combined effects of fat and muscle mass, is useful for predicting non-achievement of LDL cholesterol targets.

## 1. Introduction

Obesity is a major public health problem worldwide due to its high prevalence and heavy burden on individuals and societies. Numerous pieces of clinical and epidemiological evidence have demonstrated the strong link between obesity and cardiovascular disease (CVD) development [1], and excessive adipose tissue has been shown to worsen CVD risk factors, such as insulin resistance, abnormal glucose and lipid metabolism, hypertension, and inflammation [2,3].

The most significant contributing factor in obesity-related dyslipidemia has been identified as elevated free fatty acid (FFA) levels due to increased FFA release from adipose tissue and a reduction in plasma FFA clearance [4]. Increased FFA levels result in increased levels of triglycerides, decreased levels of high-density lipoprotein (HDL) cholesterol, and the increased presence of small, dense, low-density lipoprotein (LDL) particles, which are associated with increased risk of CVD [4,5]. Excess body fat accumulation, even in non-obese people, has been reported to be related to dyslipidemia [6]. In addition, Lee et al. demonstrated that decreased skeletal muscle mass was also associated with dyslipidemia, regardless of the presence of abdominal obesity, and suggested that insulin resistance may be associated with low muscle mass [7]. Thus, both excessive fat mass and the relative decrease in muscle mass should be considered when examining the associations between obesity and dyslipidemia.

Body mass index (BMI) is the most widely used indicator of obesity and can be measured with relative ease by both clinicians and patients. However, BMI cannot be used as a direct measure of body composition, which represents a major limitation because the same BMI can represent a variety of body compositions associated with differing health outcomes [8,9]. This limitation of BMI has led to the suggested use of other anthropometric indicators to evaluate central obesity or adiposity, such as waist circumference, waist–hip ratio, or body fat percentage [10,11]. Recently, the fat-to-muscle ratio (FMR) has been proposed as a novel indicator to assess the combined effects of fat and skeletal muscle mass. Several studies have reported that the FMR is associated with metabolic syndrome [12,13], insulin resistance [14], and nonalcoholic fatty liver disease [15]. However, the relationship between FMR and the risk of dyslipidemia is not yet known.

Maintaining an optimal LDL cholesterol level is emphasized to prevent CVD, and many international working groups have recommended the use of individualized target LDL cholesterol levels for the management of CVD based on individual CVD risk levels [16,17]. Therefore, this study aimed to investigate whether high FMR is associated with the non-achievement of LDL cholesterol targets among adults with optimal LDL cholesterol levels at baseline, using a large-sample, community-based Korean cohort observed over 12 years. Furthermore, we compared the predictive power of FMR and BMI for the non-achievement of LDL cholesterol targets.

## 2. Materials and Methods

### 2.1. Study Population

**Figure 1:**
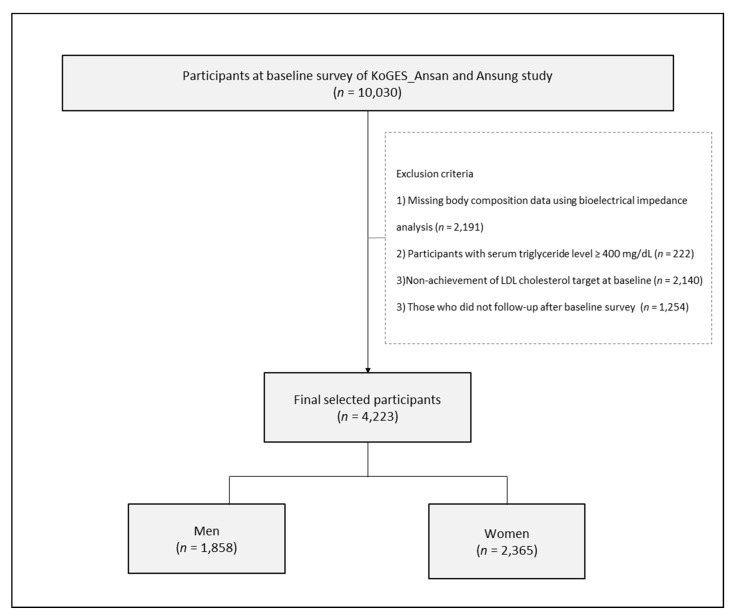
Flow chart of the study population selection process.

All data used in this study derived from the Korean Genome and Epidemiology Study (KoGES)-Ansan and Ansung study. The KoGES_Ansan and Ansung study is a longitudinal, prospective, cohort study initiated by the Korean National Institute of Health to evaluate risk factors for non-communicable diseases [18]. The survey was conducted biennially from 2001 to 2002 (baseline survey) and 2013 to 2014 (sixth follow-up). Figure 1 displays a flowchart of the study population selection process. From a total of 10,030 community-dwelling individuals aged 40–69 years who participated in the baseline survey, we excluded (1) individuals missing body composition data measured by bioelectrical impedance analysis (BIA; *n* = 2191); (2) participants with serum triglyceride level ≥ 400 mg/dL (*n* = 222); (3) participants who did not achieve the LDL cholesterol target at baseline (*n* = 2140); and (4) those who were not followed up after the baseline survey (*n* = 1254). Data from a total of 4223 participants (including 1858 men and 2365 women) were analyzed in this study.

### 2.2. Assessment of the LDL Cholesterol Target Levels Based on CVD Risk Levels

At each follow up time, participants were categorized into 4 groups based on their CVD risk levels: low-risk group, moderate-risk group, high-risk group, and very high risk group. Participants who presented with 0 or 1 major CVD risk factors were classified into the low-risk group. The moderate-risk group comprised participants with ≥2 major CVD risk factors. Participants with diabetes mellitus but without signs of target organ damage (glomerular filtration rate < 60 mL/min/1.73 m^2^, albuminuria, or the concurrence of hypertension) were categorized into the high-risk group. Participants with a prior history of coronary artery disease, ischemic stroke, or transient ischemic attack and diabetic patients with signs of target organ damage and reported current smoking at baseline were classified into the very high risk group. The major risk factors for CVD included (1) men aged ≥ 45 years and women aged ≥ 55 years; (2) systolic blood pressure (SBP) ≥ 140 mmHg, diastolic blood pressure (DBP) ≥ 90 mmHg, or current treatment with antihypertensive medications ≥ 20 days/month; (3) current smoking; (4) serum HDL cholesterol levels < 40 mg/dL; and (5) family history (parents or siblings) of premature CVD that developed < 55 years in men and < 65 years in women. Serum HDL cholesterol levels ≥ 60 mg/dL were considered to be a protective factor against CVD risk [19].

The LDL cholesterol target levels were set according to CVD risk levels as follows: <160 mg/dL for the low-risk group, <130 mg/dL for the moderate-risk group, <100 mg/dL for the high-risk group, and <70 mg/dL for the very high risk group [19]. We defined the non-achievement of an LDL cholesterol target when the LDL cholesterol level was higher than the defined LDL cholesterol target for the established CVD risk level. Participants’ CVD risk levels were assessed at each follow-up period as well as non-achievement of LDL cholesterol targets according to their CVD risk levels.

### 2.3. Assessment of Body Composition

Each participant’s body composition was analyzed using a multi-frequency BIA machine (Inbody 330; Biospace, Seoul, Korea) featuring eight tactile electrode points, which has been validated in previous studies as a reliable tool for the assessment of body composition [20,21,22]. Height (m) and weight (kg) were measured to the nearest 0.1 cm and 0.1 kg, respectively. BMI was calculated as the weight divided by height squared (kg/m^2^). A BMI greater than 25 kg/m^2^ was considered obese according to the definitions established by the Korean Society for the Study of Obesity [23]. Waist circumference (cm) was measured in the horizontal plane, midway between the iliac crest and the lowest rib. In addition, total fat mass (kg) and total skeletal muscle mass (kg) were evaluated. FMR was defined as the ratio between total fat mass and total skeletal muscle mass. Participants were classified into 3 groups according to sex-specific FMR tertiles: T1, FMR < 0.241; T2, FMR of 0.241–0.314; and T3 FMR > 0.314 in men; T1, FMR < 0.439; T2 FMR of 0.439–0.527; and T3 FMR > 0.527 in women.

### 2.4. Data Collection

After at least 30 min of rest, SBP and DBP were measured in a seated position. Mean blood pressure (MBP; mmHg) was calculated using the following equation: MBP = (SBP + 2 × DBP)/3. Alcohol intake status was classified according to whether the participant was a current drinker. Smoking status was classified according to whether the participant was a current smoker. Physical activity was assessed using metabolic equivalent of task (MET)-hours per week (METs-hr/wk). MET was obtained from the participant’s report on hours spent on sleep and 5 types of physical activities according to intensity including heavy, moderate, light, very light, and sedentary, corresponding 7 MET, 5 MET, 3 MET, 1.5 MET, and 0 MET, respectively. Total METs-hr/wk were calculated by multiplying the reported hours spent per week by the MET values that were calculated based on each type of activity. The degree of physical activity was classified into 3 categories: <7.5 MET-hr/wk, 7.5–30 MET-hr/wk, and >30 MET-hr/wk. A blood sample from each participant was collected from the antecubital vein after at least 8 h of fasting. Plasma glucose, serum insulin, total cholesterol, triglyceride, HDL cholesterol, and C-reactive protein (CRP) levels were measured using a Hitachi 7600 Analyzer (Hitachi Co., Tokyo, Japan). For participants with serum triglyceride level < 400 mg/dL, LDL cholesterol levels were calculated using the Friedewald equation: LDL cholesterol (mg/dL) = total cholesterol – HDL cholesterol – triglycerides/5 [24]. For the assessment of each participant’s diet, a 24 h dietary recall method was used. Total calorie intake (kcal/day), carbohydrate intake (g/day), fat intake (g/day), and protein intake (g/day) were calculated. Hypertension was defined using the following criteria: (1) SBP ≥ 140 mmHg, (2) DBP ≥ 90 mmHg, or (3) treatment with antihypertensive medications [25]. Diabetes mellitus was defined using the following criteria: (1) fasting plasma glucose level ≥ 126 mg/dL; (2) 2 h plasma glucose level ≥ 200 mg/dL after a 75 g oral glucose tolerance test; (3) glycosylated hemoglobin (HbA1c) ≥ 6.5%; or (4) treatment with antidiabetic medications [26]. Participants’ status of taking anti-dyslipidemic medication was obtained from a self-reported questionnaire given to each participant. Anti-dyslipidemic medication status was categorized into two groups.

### 2.5. Statistical Analysis

All data analyzed in this study are presented as the mean ± standard deviation or the median (25th percentiles, 75th percentiles) for continuous variables and as the number (percent, %) for categorical variables. To compare differences in continuous variables among the defined sex-specific FMR tertile groups, analysis of variance (ANOVA) or the Kruskal–Wallis test was used. Chi-square tests were used to compare categorical variables. A Cox proportional hazard spline curve was used to verify the linearity of the relationship between FMR and the incidence of non-achievement of LDL cholesterol targets. Kaplan–Meier curves were used to assess the cumulative incidence of non-achievement of LDL cholesterol targets according to the sex-specific FMR tertiles. The log-rank test was used to assess among-group comparisons of the distribution of the cumulative incidence of non-achievement of LDL cholesterol targets. Cox proportional hazard regression analysis was used to calculate the hazard ratio (HR) with a 95% confidence interval (CI) for incident non-achievement of LDL cholesterol targets in the T2 and T3 tertiles vs. the referent T1 tertile according to sex. Generalized estimating equation (GEE) models were generated to determine the relationship between baseline FMR and the longitudinal proportion of non-achievement of LDL cholesterol targets according to the sex-specific FMR tertiles. Receiver operating characteristic (ROC) curves were used to compare the discriminative power of FMR and BMI to predict the non-achievement of LDL cholesterol targets using the area under the ROC curve (AUC). All statistical analyses were conducted using SPSS statistical software (version 25.0; SPSS Inc., Chicago, IL, USA), SAS statistical software (version 9.4; SAS Institute Inc., Cary, NC, USA), and R (Version 4.0.3; R Foundation for Statistical Computing, Vienna, Austria). *p* < 0.05 was considered statistically significant.

## 3. Results

### 3.1. Clinical Characteristics of the Study Population

The baseline characteristics of the study population are represented in Table 1. For both men and women, mean age, BMI, MBP, plasma glucose, serum total cholesterol, LDL cholesterol levels, median serum triglyceride levels, and the proportion of obese participants and people with hypertension were significantly increased in the sex-specific T3 tertile compared with the T1 tertile. The mean value of serum HDL cholesterol levels and the proportion of participants who exercised > 30 METs-h/week were significantly decreased in the sex-specific T3 tertile compared with the T1 tertile for both men and women. In men but not women, the proportion of current smokers significantly decreased in the T3 tertile compared with the T1 tertile. In women but not men, the proportion of current drinkers significantly decreased in the T3 tertile compared with the T1 tertile.

### 3.2. Longitudinal Relationship between FMR and the Incident Non-Achievement of LDL Cholesterol Targets

**Figure 2:**
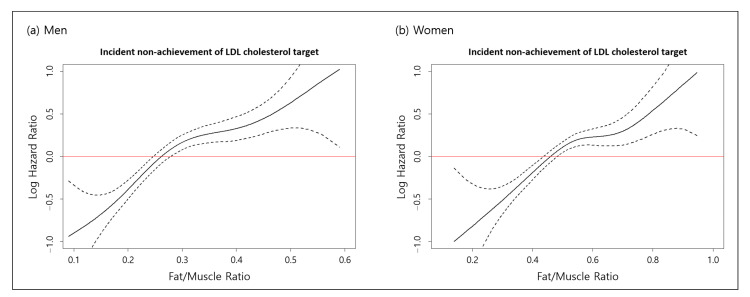
Relationship between the incidence of non-achievement of LDL cholesterol targets and FMR in (**a**) men and (**b**) women. Graphs showing the incidence of non-achievement of LDL cholesterol targets according to FMR (solid lines), with 95% CI bands (broken lines) according to the Cox proportional hazard spline curve. Abbreviations: LDL, low-density lipoprotein; FMR, fat-to-muscle ratio; CI, confidence interval.

Figure 2 shows the linear relationship between FMR and the incident non-achievement of LDL cholesterol targets using Cox proportional hazard spline curves. As the continuous FMR values increased, the risk of incident non-achievement of LDL cholesterol targets increased in both men and women.

**Figure 3:**
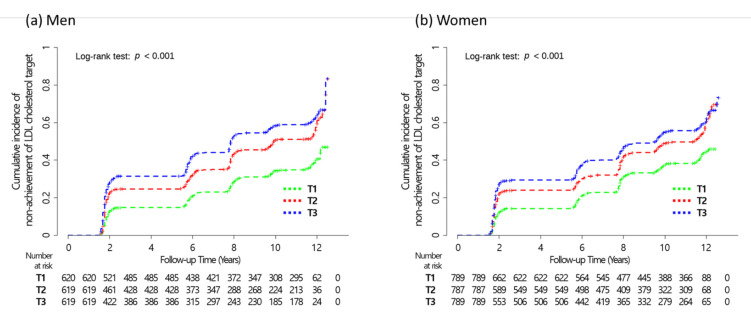
Kaplan–Meier curves for the cumulative incidence of non-achievement of LDL cholesterol targets according to sex-specific FMR tertile groups in (**a**) men and (**b**) women. Abbreviations: LDL, low-density lipoprotein; FMR, fat-to-muscle ratio; T, tertile.

Figure 3 shows Kaplan–Meier curves for the cumulative incidence of non-achievement of LDL cholesterol targets according to the sex-specific FMR tertiles. The cumulative incident non-achievement of LDL cholesterol targets was significantly highest in the T3 tertile, followed by the T2 and T1 tertiles in both men and women (*p* for log-rank tests < 0.001 for both men and women).

Table 2 presents the results of a Cox proportional hazard analysis for the incidence of non-achievement of LDL cholesterol targets according to the sex-specific FMR tertiles. The HRs and 95% CIs for the incidence of non-achievement of LDL cholesterol targets in the T2 and T3 tertiles vs. the referent T1 tertile were 1.65 (1.39–1.97) and 2.08 (1.75–2.47) in men and 1.49 (1.28–1.73) and 1.72 (1.49–2.00) in women, respectively. After adjusting for age, obesity, current smoking, current drinking, physical activity, total caloric intake, MBP, plasma glucose, serum CRP, baseline serum LDL cholesterol levels, and taking anti-dyslipidemic medication at baseline, the adjusted HRs and 95% CI for the incidence of non-achievement of LDL cholesterol targets for the T2 and T3 tertiles vs. the referent T1 tertile were 1.56 (1.29–1.90) and 1.86 (1.47–2.31) in men and 1.40 (1.18–1.66) and 1.31 (1.06–1.62) in women, respectively. 

### 3.3. Proportions of Non-Achievement of LDL Cholesterol Targets in the Sex-specific FMR Tertile Groups during the Follow-up Period

Table 3 represents a comparison of the estimated proportions of non-achievement of LDL cholesterol targets among the sex-specific FMR tertile groups during the follow-up periods using GEE models. In both the overall and post hoc analyses, the estimated proportion of people with the non-achievement of LDL cholesterol targets in the T3 tertile remained significantly higher than that in the T1 tertile during all follow-up periods for both men and women, except for the sixth follow-up period in women. The group-by-time interactions were significant for both men and women.

### 3.4. Comparison of the Predictive Powers of FMR and BMI for the Non-Achievement of LDL Cholesterol Targets

**Figure 4:**
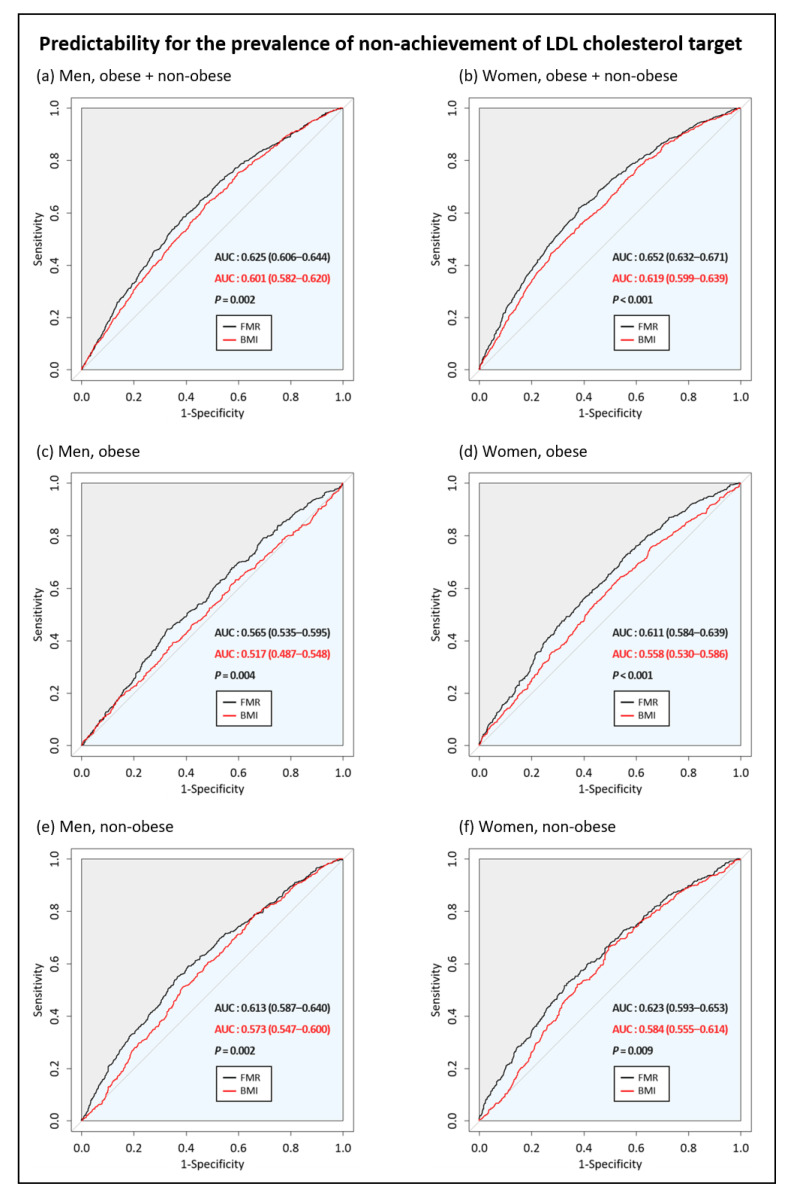
Comparison of the predictive powers of FMR and BMI for non-achievement of LDL cholesterol targets in (**a**) men, (**b**) women, (**c**) obese men, (**d**) obese women, (**e**) non-obese men, and (**f**) non-obese women. Abbreviations: FMR, fat-to-muscle ratio; BMI, body mass index; LDL, low-density lipoprotein.

Figure 4 compares the predictive powers of FMR and BMI for the non-achievement of LDL cholesterol targets. The AUCs for FMR and BMI were 0.625 and 0.601 in men and 0.652 and 0.619 in women, respectively. The predictive power of FMR was significantly higher than that of BMI for both men and women (*p* = 0.002 in men; *p* < 0.001 in women). When comparing predictive powers between obese and non-obese subjects, we found that that the predictive powers of FMR were significantly higher than those of BMI for both men and women (*p* = 0.004 in obese men; *p* = 0.002 in obese women; *p* < 0.001 in non-obese men; *p* = 0.009 in non-obese women).

## 4. Discussion

We examined the longitudinal relationship between FMR and the incidence of non-achievement of LDL cholesterol targets. Our results showed an increasing trend in the incidence of non-achievement of LDL cholesterol targets with higher FMR based on Cox proportional hazard spline curves. We also found that the estimated proportion of people with incident non-achievement of LDL cholesterol targets was significantly higher in the highest FMR tertile compared with the lowest tertile during almost all follow-up periods in both men and women. These significant results remained even considering the higher proportion of participants who took anti-dyslipidemic medications of T2 or T3 vs. T1 during the period in both men and women. The number of participants taking anti-dyslipidemic medications according to the tertiles of fat-to-muscle ratio are shown in Table S1. In addition, the predictive power of FMR for the incidence of non-achievement of LDL cholesterol targets was significantly higher than that of BMI among total, obese, and non-obese subjects.

To the best of our knowledge, this study is the first longitudinal study to examine the association between FMR and the incidence of non-achievement of LDL cholesterol targets. Seo et al. revealed that high FMR was significantly associated with the prevalence of metabolic syndrome and insulin resistance and determined sex-specific optimal FMR cutoff values to predict metabolic syndrome in a Korean population [14]. Similarly, Chen et al. showed significant associations between FMR and metabolic syndrome, diabetes mellitus, and hypertension, suggesting the usefulness of FMR as a predictive index for cardio-metabolic risks [27]. Our results are consistent with previous studies showing a strong relationship between FMR and CVD risk [14,27] and are strengthened by data from a longitudinal, prospective cohort study.

Several possible mechanisms support our results. Accumulated evidence has demonstrated a relationship between increased fat mass and dyslipidemia. The most likely contributing factor for adiposity-related dyslipidemia is uncontrolled fatty acid lipolysis, leading to the increased delivery of FFA to the liver, upregulating triglyceride synthesis, and exacerbating dyslipidemia [4,5]. In addition, excessive body fat increases the secretion of pro-inflammatory adipokines, such as tumor necrosis factor-α and serum amyloid A, by both adipocytes and adipose tissue-associated macrophages [28,29]. Conversely, the secretion of anti-inflammatory adipokines may be decreased. This imbalance between pro- and anti-inflammatory adipokines may lead to impaired insulin sensitivity in adipose tissue, increasing the concentrations of FFA and promoting dyslipidemia [28,29]. Although the direct mechanisms underlying the relationship between muscle mass and dyslipidemia remain relatively unclear, several plausible explanations exist. Skeletal muscle is considered an important insulin-responsive endocrine organ, and decreased muscle mass contributes to impaired glycemic control and insulin resistance [30], which could contribute to the development of atherogenic dyslipidemia [31]. The accumulation of intra- and inter-muscular adipose tissue, accompanied by decreased muscle mass, can induce muscle inflammation and negatively regulate myocyte metabolism, leading to insulin resistance [32,33].

Interestingly, we observed a small difference in sex in the association between FMR and the non-achievement of LDL cholesterol targets. In the GEE models, the estimated proportions of people with incident non-achievement of LDL cholesterol targets in the T2 and T3 tertiles showed no significant differences from the proportions in the post hoc analyses for both men and women. However, the estimated proportions of incident non-achievement of LDL cholesterol targets in the T2 tertile at the fifth follow-up and in the T3 tertile at the sixth follow-up were not significantly different from those in the T1 tertile among women. Although the reasons for these differences remain unclear, sexual dimorphism of body fat distribution [34] and changes in the body composition after menopause due to sex hormones in women [35] might affect this result.

Our second aim was to compare the predictive powers between FMR and BMI for the incidence of non-achievement of LDL cholesterol targets. Whether obesity or body fat indicators other than BMI can present better predictive power for CVD risk than BMI remains controversial. Several studies have shown that waist circumference and body fat percentage are more strongly associated with CVD risk factors than BMI [36,37]. For example, Byambasukh et al. identified that body fat percentage measured by BIA was independently associated with incident CVD events, and the predictive value of body fat percentage was superior to both BMI and waist circumference in a prospective cohort study [37]. Conversely, other studies have shown that BMI, which is a simple and inexpensive measure, remains a better predictor of CVD risk than other obesity indicators [38]. These inconsistent findings may be associated with differences in clinical characteristics or inherent shortcomings of each measure, such as the BMI-associated misclassification of individuals with high muscle mass as obese [39] and the underestimation of body fat percentages measured by BIA [40]. In this study, we found that the predictive power of FMR for the incidence of non-achievement of LDL cholesterol targets was higher than that of BMI in both men and women. When analyzed by subgroups of obese and non-obese men and women, FMR was a better predictor of dyslipidemia than BMI. Our findings suggest the potential for using FMR as an obesity indicator to compensate for the shortcomings of BMI, which does not adequately reflect body composition.

This study has several limitations. First, we did not consider the effects of changes during the follow-up period, including any changes in fat and muscle mass that would affect FMR levels or changes in other covariates. The consideration of changes in body composition and other covariates during the follow-up period will be incorporated into the next study. Second, fat mass and skeletal muscle mass were measured by BIA instead of dual-energy X-ray absorptiometry, which is considered a more reliable method for body composition assessment [41]. However, BIA is a popular, inexpensive, non-invasive, and validated measurement. Third, we could not assess the contributions of fat distribution to the association between FMR and the non-achievement of LDL cholesterol targets. FMR was calculated using only total fat mass and total skeletal muscle mass. Evidence has suggested the relative importance of visceral fat rather than subcutaneous fat to increased CVD risk [42]. Fourth, some major CVD risk factors were not completely assessed due to a lack of information regarding the incidence of carotid artery stenosis, peripheral artery disease, and abdominal aortic aneurysm. Fifth, serum LDL cholesterol level was not measured directly. Although we excluded participants whose serum triglyceride level was ≥400 mg/dL, the Friedewald equation tends to underestimate participants’ LDL cholesterol level in those with serum triglyceride level of 200–399 mg/dL [43]. In addition, the number of participants taking anti-dyslipidemic medications was low during the period. Prior guidelines on the management of dyslipidemia, which set the LDL cholesterol level target < 100 mg/dL in coronary heart disease (CHD), and CHD risk equivalent made doctors prescribe anti-dyslipidemic medications less often than now [44,45]. In the baseline survey, participants may be relatively unaware of whether they were taking anti-dyslipidemic medications. There is also lack of information about the type of anti-dyslipidemic medications such as statin in KoGES data. Therefore, information bias should be taken into account in this study. Finally, selection bias should be considered due to the relatively high proportion of missing values, especially associated with the lack of body composition data using BIA. Therefore, these results should be interpreted with caution. Despite these weaknesses, our study has several strengths. To the best of our knowledge, this study is the first to examine the association between FMR and incidence of non-achievement of LDL cholesterol targets based on individual CVD risk levels, using a large, prospective cohort study. Moreover, we compared the predictive power of FMR with that of BMI to demonstrate the usefulness of FMR.

## 5. Conclusions

In conclusion, high FMR is significantly associated with an increased risk of non-achievement of LDL cholesterol targets. Furthermore, FMR is a better predictor of the non-achievement of LDL cholesterol targets than BMI. Therefore, we suggest that FMR, which reflects the combined effects of fat and muscle mass, can serve as a novel indicator for the possibility of maintaining optimal LDL cholesterol levels according to individualized CVD risk.

## Figures and Tables

**Table 1:** Baseline characteristics of the study population.

	Men				Women			
Fat-to-Muscle Ratio	T1 (<0.241)	T2 (0.241–0.314)	T3 (>0.314)	*p*	T1 (<0.439)	T2 (0.439–0.527)	T3 (>0.527)	*p*
Number, n	620	619	619		789	787	789	
Age, years	50.6 ± 8.8	50.3 ± 8.5	51.4 ± 8.6	0.096	49.0 ± 8.4	49.7 ± 8.2	51.7 ± 8.7	<0.001
BMI, kg/m^2^	21.6 ± 2.2	23.9 ± 2.0	26.3 ± 2.3	<0.001	21.9 ± 2.0	24.3 ± 1.9	27.3 ± 2.7	<0.001
Obese, n (%)	35 (5.6%)	175 (28.3%)	440 (71.1%)	<0.001	43 (5.4%)	280 (35.6%)	640 (81.1%)	<0.001
Mean blood pressure, mmHg	93.7 ± 12.0	96.2 ± 11.2	98.8 ± 11.8	<0.001	89.3 ± 12.2	91.9 ± 12.9	95.4 ± 12.7	<0.001
Glucose, mg/dL	82.6 ± 9.7	85.3 ± 14.5	86.6 ± 11.9	<0.001	79.9 ± 7.3	80.8 ± 10.5	82.2 ± 11.5	<0.001
Total cholesterol, mg/dL	171.3 ± 27.7	178.7 ± 26.7	183.2 ± 26.2	<0.001	174.3 ± 27.4	179.7 ± 28.0	183.7 ± 27.9	<0.001
Triglyceride, mg/dL	111.0 [87.0; 146.5]	142.0 [108.5; 194.0]	176.0 [128.0; 236.5]	<0.001	99.0 [80.0; 131.0]	119.0 [91.0; 160.0]	128.0 [99.0; 177.0]	<0.001
HDL cholesterol, mg/dL	47.7 ± 11.2	43.0 ± 9.1	41.3 ± 8.6	<0.001	48.7 ± 10.6	45.4 ± 9.9	45.0 ± 9.4	<0.001
LDL cholesterol, mg/dL	98.5 ± 25.6	103.9 ± 26.0	104.9 ± 24.8	<0.001	103.2 ± 23.5	107.2 ± 24.2	109.4 ± 24.7	<0.001
CRP, mg/dL	0.11 [0.04; 0.19]	0.14 [0.07; 0.24]	0.16 [0.08; 0.27]	<0.001	0.09 [0.03; 0.17]	0.12 [0.05; 0.21]	0.15 [0.08; 0.27]	<0.001
Current smokier, n (%)	325 (52.7%)	256 (41.6%)	232 (37.8%)	<0.001	30 (3.9%)	15 (1.9%)	21 (2.7%)	0.070
Current drinker, n (%)	437 (70.8%)	458 (74.4%)	444 (72.5%)	0.382	249 (31.8%)	222 (28.6%)	206 (26.2%)	0.049
Physical activity, n (%)				<0.001				0.007
<7.5 METs-h/week	34 (5.8%)	38 (6.4%)	29 (4.8%)		59 (7.8%)	71 (9.3%)	91 (12.0%)	
7.5–30 METs-h/week	309 (53.0%)	394 (66.7%)	419 (69.7%)		497 (65.4%)	506 (66.3%)	510 (67.5%)	
>30 METs-h/week	240 (41.2%)	159 (26.9%)	153 (25.5%)		204 (26.8%)	186 (24.4%)	155 (20.5%)	
Daily caloric intake, kcal/day	2016.1 ± 656.5	2020.3 ± 603.3	1998.0 ± 704.9	0.632	1914.9 ± 726.6	1901.1 ± 693.3	1897.0 ± 715.8	0.623
Daily protein intake, g/day	68.3 ± 27.4	69.4 ± 25.3	68.4 ± 28.1	0.997	64.8 ± 29.6	65.4 ± 33.9	63.7 ± 29.1	0.512
Daily fat intake, g/day	35.5 ± 20.6	36.2 ± 18.3	34.8 ± 19.7	0.552	31.3 ± 19.6	30.9 ± 22.1	29.5 ± 20.5	0.093
Daily carbohydrate intake, g/day	350.3 ± 108.2	349.0 ± 101.5	347.4 ± 117.3	0.654	339.5 ± 125.3	336.1 ± 112.2	340.1 ± 124.2	0.927
Taking anti-dyslipidemic medication, n (%)	0 (0.0%)	3 (1.2%)	3 (1.1%)	0.304	0 (0.0%)	1 (0.2%)	3 (0.7%)	0.238
Hypertension, n (%)	169 (27.3%)	188 (30.4%)	270 (43.6%)	< 0.001	135 (17.1%)	179 (22.7%)	270 (34.2%)	<0.001
Diabetes mellitus, n (%)	17 (2.7%)	18 (2.9%)	14 (2.3%)	0.762	10 (1.3%)	16 (2.0%)	16 (2.0%)	0.416

Data are presented as mean ±standard deviations and median (interquartile range) or number (%). *p*-values were derived from Student’s t-test for continuous variables and the Chi-square test for categorical variables. *p* < 0.05 was considered statistically significant. Abbreviations: KoGES, Korean Genome and Epidemiology Study; BMI, body mass index; HDL, high-density lipoprotein; LDL, low-density lipoprotein; CRP, C-reactive protein; METs, metabolic equivalent of tasks. T, Tertile.

**Table 2:** HR with 95% CI for the incident non-achievement of LDL cholesterol targets according to the sex-specific tertiles of fat-to-muscle ratio.

	Fat/Muscle Ratio						
	T1	T2			T3		
		HR	95% CI	*p*	HR	95% CI	*p*
Men							
Unadjusted	1 (reference)	1.65	1.39–1.97	<0.001	2.08	1.75–2.47	<0.001
Model 1	1 (reference)	1.67	1.39–2.02	<0.001	2.14	1.73–2.65	<0.001
Model 2	1 (reference)	1.64	1.36–1.98	<0.001	2.05	1.65–2.54	<0.001
Model 3	1 (reference)	1.56	1.29–1.90	<0.001	1.86	1.47–2.31	<0.001
Women							
Unadjusted	1 (reference)	1.49	1.28–1.73	<0.001	1.72	1.49–2.00	<0.001
Model 1	1 (reference)	1.42	1.21–1.67	<0.001	1.43	1.17–1.75	<0.001
Model 2	1 (reference)	1.40	1.19–1.65	<0.001	1.42	1.16–1.74	<0.001
Model 3	1 (reference)	1.40	1.18–1.66	<0.001	1.31	1.06–1.62	0.011

Model 1: Adjusted for age, obesity, current smoker, current drinker, physical activity, and total caloric intake. Model 2: Adjusted for all variables used in Model 1 plus mean blood pressure, plasma glucose, and serum CRP level. Model 3: Adjusted for all variables used in Model 2 plus baseline serum LDL cholesterol level, and taking anti-dyslipidemic medication at baseline. Abbreviations: HR, hazard ratio; CI, confidence interval; LDL, low-density lipoprotein; CRP, C-reactive protein; T, tertile.

**Table 3:** Generalized estimating equation models predicting the effects of time on the proportions of non-achievement of LDL cholesterol targets according to the sex-specific tertiles of fat-to-muscle ratio.

	Fat/Muscle Ratio						
	T1	T2	T3				
	Estimated Proportion, % (SE)	Estimated Proportion, % (SE)	Estimated Proportion, % (SE)	Overall *p*	Post hoc *p* T2 vs. T1	Post hoc *p* T3 vs. T1	Post hoc *p* T3 vs. T2
Men							
1st f/u	15.0 (1.5)	25.1 (1.8)	32.1 (1.9)	group: *p* < 0.001 time: *p* < 0.001 group-by-time: *p* = 0.002	< 0.001	< 0.001	0.008
2nd f/u	8.3 (1.2)	13.5 (1.5)	15.7 (1.6)		0.007	< 0.001	0.330
3rd f/u	17.4 (1.7)	24.8 (2.0)	30.1 (2.1)		< 0.001	< 0.001	0.062
4th f/u	20.0 (1.8)	27.9 (2.0)	34.8 (2.2)		0.005	< 0.001	0.020
5th f/u	12.1 (1.6)	18.3 (1.8)	24.0 (2.0)		0.010	< 0.001	0.035
6th f/u	14.9 (1.7)	23.6 (2.0)	24.3 (2.1)		0.001	< 0.001	0.811
Women							
1st f/u	14.8 (1.3)	24.9 (1.6)	30.4 (1.7)	group: *p* < 0.001 time: *p* < 0.001 group-by-time: *p* < 0.001	< 0.001	< 0.001	0.018
2nd f/u	5.1 (0.9)	11.8 (1.3)	14.7 (1.4)		< 0.001	< 0.001	0.122
3rd f/u	15.8 (1.5)	22.2 (1.7)	27.2 (1.8)		0.005	< 0.001	0.041
4th f/u	22.4 (1.7)	32.2 (1.9)	31.4 (1.9)		< 0.001	< 0.001	0.752
5th f/u	19.9 (1.6)	21.2 (1.7)	24.9 (1.8)		0.589	0.042	0.139
6th f/u	17.8 (1.6)	25.5 (1.8)	22.0 (1.8)		0.001	0.075	0.175

Abbreviations: LDL, low-density lipoprotein; SE, standard error; T, tertile.

## Data Availability

Data in this study were from the Korean Genome and Epidemiology Study (KoGES; 4851-302), National Institute of Health, Korea Disease Control and Prevention Agency, Republic of Korea. The dataset used in this study can be provided after review and evaluation of the research plan by the Korea Centers for Disease Control and Prevention (http://www.cdc.go.kr/CDC/eng/main.jsp).

## Supplementary Materials

The following are available online at https://www.mdpi.com/article/10.3390/jcdd8080096/s1. Table S1: Number of participants taking anti-dyslipidemic medications according to the tertiles of fat-to-muscle ratio.
