# Supplementary material for: Fat-to-Muscle Ratios and the Non-Achievement of LDL Cholesterol Targets: Analysis of the Korean Genome and Epidemiology Study
Source: J Cardiovasc Dev Dis. 2021 Aug 12;8(8):96. doi: 10.3390/jcdd8080096 (PMC8396939; doi:10.3390/jcdd8080096)
Supplement: Supplementary file 1 [file jcdd-08-00096-s001.zip › jcdd-1323801-supplementary.pdf]

Table S1. Number of participants taking anti-dyslipidemic medications according to the tertiles of fat-to-muscle ratio.

	Men				Women			
Fat-to-muscle ratio	T1 (< 0.241)	T2 (0.242–0.314)	T3 (> 0.314)	*p*	T1 (< 0.439)	T2 (0.439–0.527)	T3 (> 0.527)	*p*
Participants taking anti-dyslipidemic medications, n (%)								
Baseline	0 (0.0%)	3 (1.2%)	3 (1.1%)	0.304	0 (0.0%)	1 (0.2%)	3 (0.7%)	0.238
1st f/u	1 (0.8%)	0 (0.0%)	1 (0.6%)	0.608	0 (0.0%)	2 (0.9%)	8 (2.6%)	0.032
2nd f/u	3 (0.6%)	2 (0.4%)	6 (1.2%)	0.307	4 (0.6%)	1 (0.2%)	10 (1.6%)	0.014
3rd f/u	5 (1.1%)	4 (0.8%)	7 (1.4%)	0.689	3 (0.5%)	12 (2.0%)	9 (1.5%)	0.072
4th f/u	2 (0.4%)	5 (1.0%)	17 (3.6%)	<0.001	15 (2.4%)	24 (3.9%)	29 (4.8%)	0.070
5th f/u	16 (3.6%)	11 (2.5%)	28 (6.1%)	0.022	33 (5.4%)	46 (7.9%)	44 (7.8%)	0.163
6th f/u	21 (4.8%)	26 (6.0%)	41 (9.5%)	0.018	57 (9.7%)	89 (15.1%)	80 (14.8%)	0.008

p derived from chi-square test or Fisher's exact test.

p <0.05 considered statistically significant.
